# Supplementary material for: Cdc42 regulates cytokine expression and trafficking in bronchial epithelial cells
Source: Front Immunol. 2022 Dec 23;13:1069499. doi: 10.3389/fimmu.2022.1069499 (PMC9816864; doi:10.3389/fimmu.2022.1069499)
Supplement: Supplementary file 1 [file DataSheet_1.pdf]

Supplemental Table 1

BEAS-2B cytokine secretion levels (pg/mL) after 8 h of stimulation

	Replication 1					Replication 2					Replication 3				
	Resting	polyI:C	CE	TNF-α	TNF-α + ML141	Resting	polyI:C	CE	TNF-α	TNF-α + ML141	Resting	polyI:C	CE	TNF-α	TNF-α + ML141
**GM-CSF**	N/A	N/A	N/A	18.2	N/A	N/A	N/A	N/A	N/A	N/A	N/A	N/A	N/A	20.7	2.19
**IFN-γ**	N/A	N/A	N/A	N/A	N/A	N/A	N/A	N/A	N/A	N/A	N/A	N/A	N/A	0.3	N/A
**IL-1β**	N/A	N/A	N/A	0.43	N/A	1.7	1.1	0.8	2.3	2.6	1.1	1.9	1.3	2.1	1.9
**IL-Ra**	N/A	N/A	N/A	N/A	N/A	N/A	N/A	N/A	N/A	N/A	0.5	0.6	0.6	0.7	0.6
**IL-2**	N/A	N/A	N/A	N/A	N/A	N/A	N/A	N/A	N/A	N/A	N/A	N/A	N/A	0.2	0.2
**IL-4**	N/A	N/A	N/A	N/A	N/A	N/A	N/A	N/A	N/A	N/A	N/A	N/A	N/A	N/A	N/A
**IL-5**	N/A	N/A	N/A	N/A	N/A	N/A	N/A	N/A	N/A	N/A	N/A	N/A	N/A	N/A	N/A
**IL-6**	2.6	4.6	4.3	48.2	83.6	0.3	0.3	0.4	9.9	10.5	2.4	7.5	5.3	31.3	65.6
**IL-8**	41.5	70.2	56.0	693.0	599.3	5.3	6.7	2.3	85.3	79.6	68.4	135.5	103.7	1993.1	1348.8
**IL-10**	N/A	N/A	N/A	N/A	N/A	N/A	N/A	N/A	N/A	N/A	N/A	N/A	N/A	N/A	N/A
**IL-12p40**	N/A	N/A	N/A	N/A	N/A	N/A	N/A	N/A	1.3	1.6	3.4	2.3	3.7	5.5	4.4
**IL-12p70**	N/A	N/A	N/A	N/A	N/A	N/A	N/A	N/A	N/A	N/A	N/A	N/A	N/A	N/A	N/A
**IL-13**	N/A	N/A	N/A	3.57	N/A	N/A	N/A	N/A	N/A	N/A	N/A	N/A	N/A	1.31	N/A
**MCP-1**	90.1	116.9	120.8	1625.0	255.9	7.7	8.1	N/A	144.8	37.5	76.4	78.1	126.5	1949.1	332.7
**TNF-α**	N/A	N/A	N/A	1584.5	1211.2	N/A	N/A	N/A	1046.5	1172.2	N/A	N/A	N/A	7875.1	7656.5

Human cytokine multiplex analysis (HDF15) performed by EveTechnologies™. BEAS-2B cells were stimulated 8 h with 10 µg/mL poly(I:C), 20 µg/mL cockroach extract (CE) or 10 ng/mL TNF-α. ML141 was used at 20 µM and added to cells one hour prior to stimulation. Data represents three independent experiments. N/A – not detected (secretion levels below detection range).

Supplemental Table 2

Cytokine secretion levels (pg/mL) from BEAS-2B Cdc42 KD and control cells after 8 h of TNF-α stimulation

	Replication 1		Replication 2		Replication 3	
	ShCtrl	Cdc42-shRNA 3	ShCtrl	Cdc42-shRNA 3	ShCtrl	Cdc42-shRNA 3
GM-CSF	6.02	N/A	0.50	N/A	9.89	N/A
IFN-γ	1.24	1.17	0.91	1.25	1.51	1.03
IL-1β	2.58	2.75	2.46	1.87	3.27	2.11
IL-Ra	0.39	0.49	0.38	0.43	0.45	0.38
IL-2	0.14	0.23	0.16	0.17	0.22	0.17
IL-4	0.08	0.05	0.06	0.05	0.06	0.06
IL-5	0.06	0.07	0.05	0.06	0.06	0.05
IL-6	17.28	15.98	13.25	15.76	22.61	15.53
IL-8	1509.97	1413.76	884.23	1470.22	1883.86	1451.87
IL-10	0.38	0.35	0.30	0.32	0.44	0.34
IL-12p40	4.58	5.25	2.87	4.96	5.16	4.96
IL-12p70	0.33	0.32	0.28	0.25	0.44	0.24
IL-13	5.75	4.98	4.20	5.90	6.50	4.20
MCP-1	50000	1856.46	50000	796.57	50000	804.62
TNF-α	100000	100000	100000	100000	100000	100000

Human cytokine multiplex analysis (HDF15) performed by EveTechnologies™. BEAS-2B Cdc42 KD or scrambled control cells, were stimulated 8 h with 10 ng/mL TNF-α. Data represents three independent experiments. N/A, not detected (secretion levels below detection range); values in orange color, extrapolated secretion levels based on standard curve (above detection range).

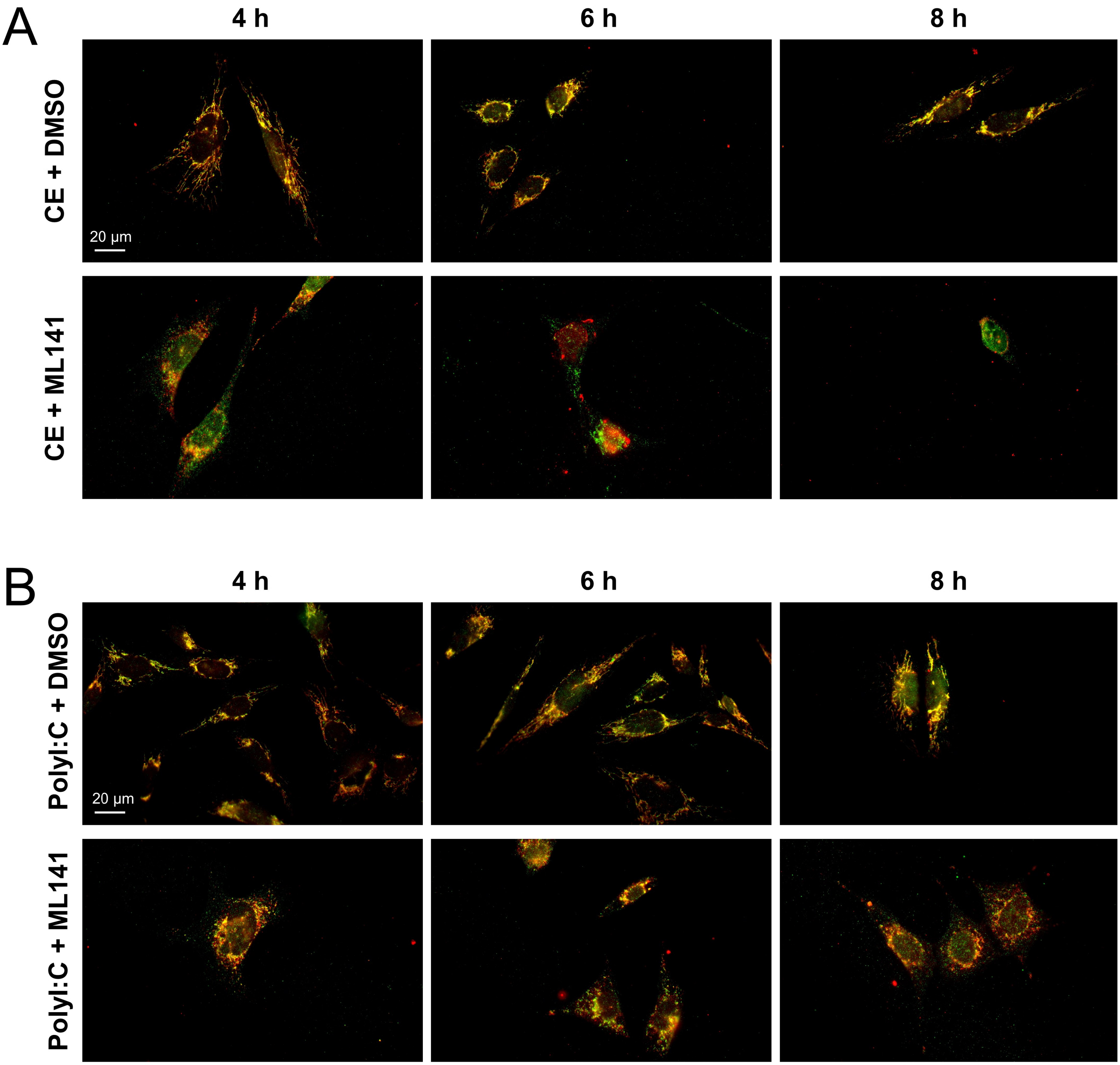

Figure S1 IL-8 and TSLP show post-Golgi tubule staining patterns that are affected by Cdc42 inhibition. BEAS-2B cells were pretreated with vehicle (DMSO) or 20 µM ML141 for 1 h, then stimulated with 20 µg/ml cockroach extract (CE) **(A)** or 10 µg/ml poly(I:C) **(B)** for 4 h, 6 h and 8 h. Cells were then fixed and stained with IL-8 (*red*) and TSLP (*green*) antibodies. ML141 affects the post-Golgi tubule staining pattern which is most prominently observed at 4 h of stimulation.

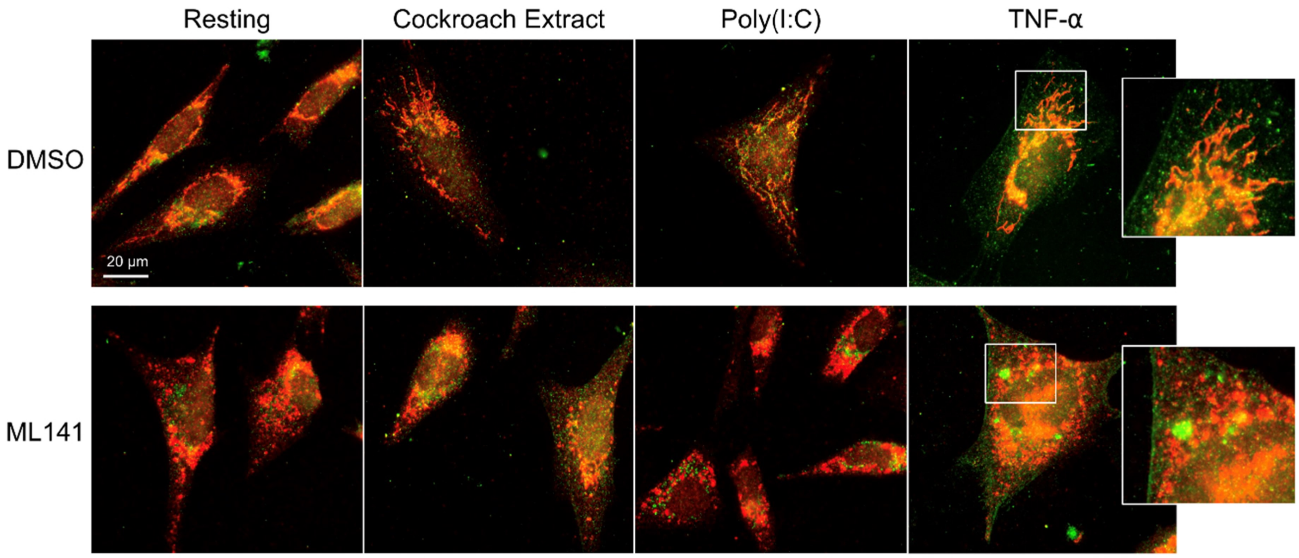

Figure S2 IL-8 and IL-1β show non-overlapping staining patterns and are differentially affected by Cdc42 inhibition. BEAS-2B cells were pretreated with 20 μM ML141 or vehicle (DMSO) for 1 h, then stimulated with 10 ng/ml TNF-α for 4 h. Cells were then fixed and stained with IL-8 (*red*) and IL-1β (*green*) antibodies. Zoomed panels show the two cytokines display different characteristic staining patterns. ML141 affects the IL-8 staining pattern but does not affect IL-1β staining.

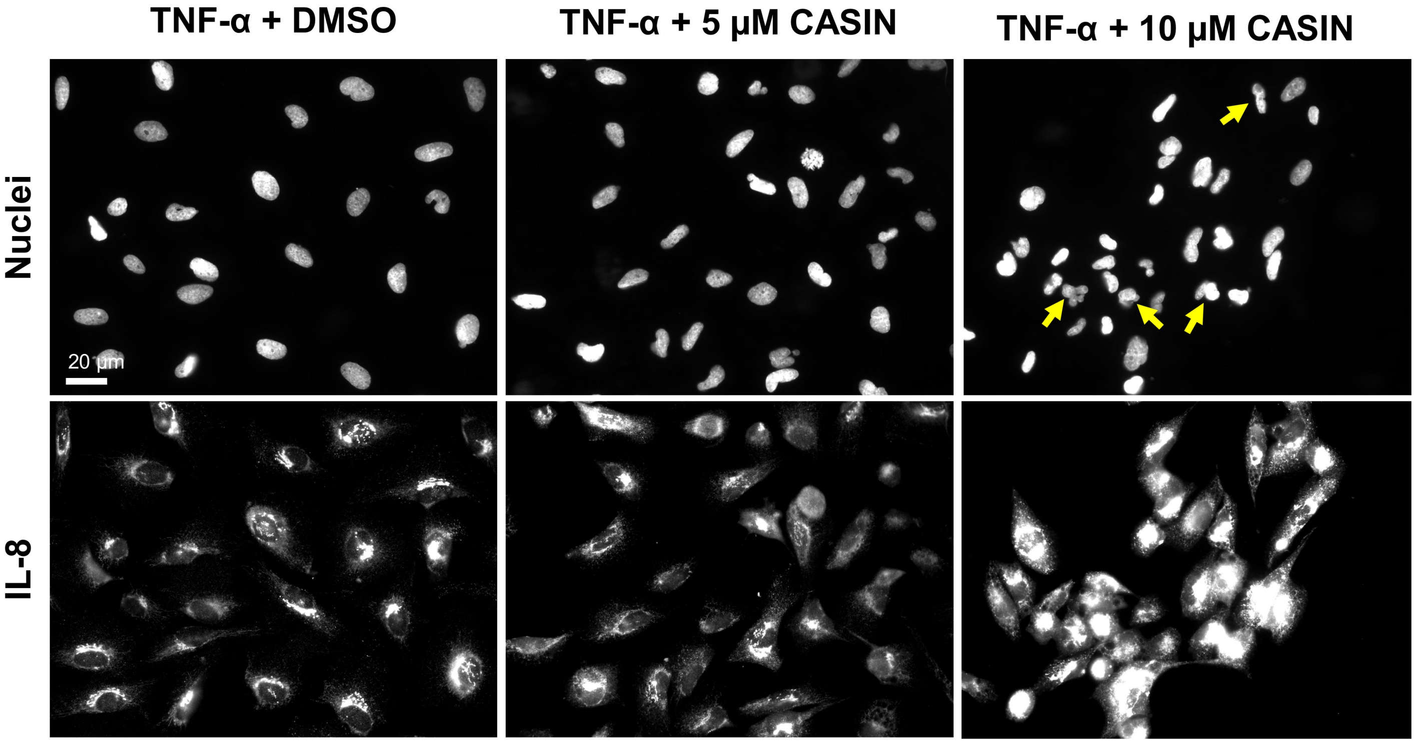

Figure S3 Effect of the Cdc42 inhibitor, CASIN, on BEAS-2B cells. BEAS2B cells were pre-treated with vehicle (DMSO) or CASIN at 5 µM or 10 µM for 1 h. Cells were then stimulated with 10 ng/ml TNF-α for 4 h. Cells were then fixed and stained for immunofluorescence with IL-8 antibodies to assess cytokine trafficking and DAPI to assess nuclear fragmentation, which indicates apoptotic cell death. 10 µM CASIN distrupted IL-8 post-Golgi staining of tubules however, some cells also showed nuclear fragmentation (*yellow arrows*) indicating a toxic effect. Note that at 20 µM CASIN, no cells remained to image likely due to a highly toxic effect resulting in massive cell death.

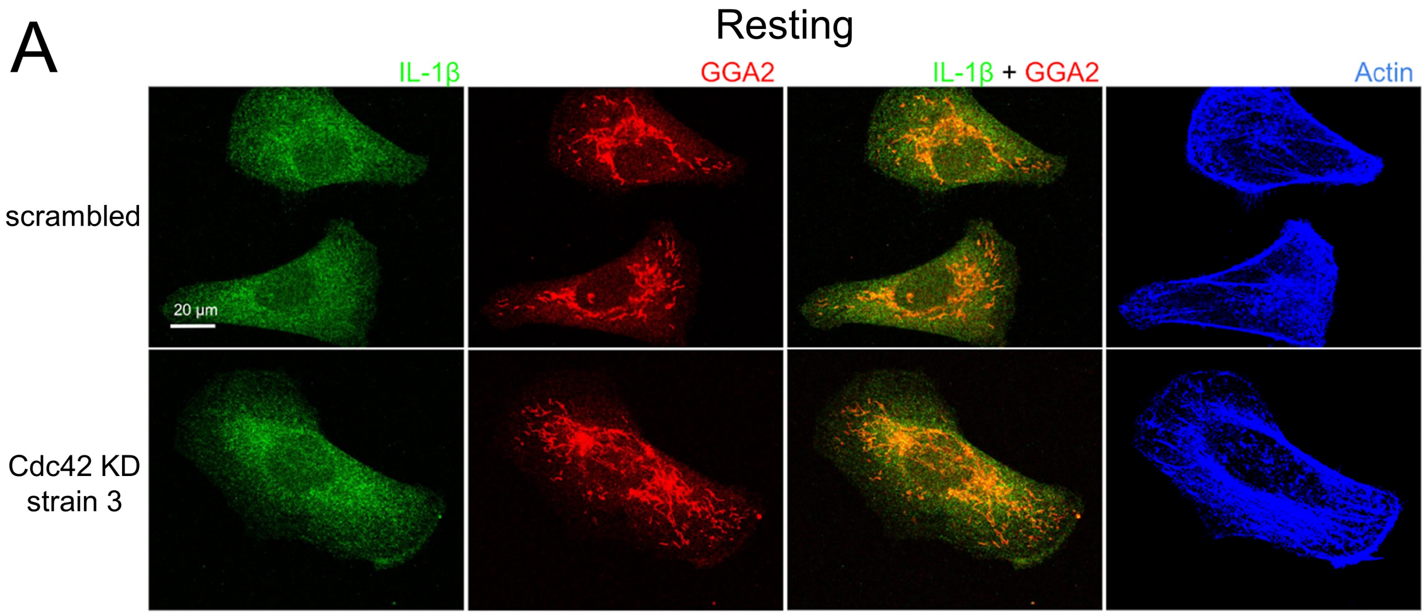

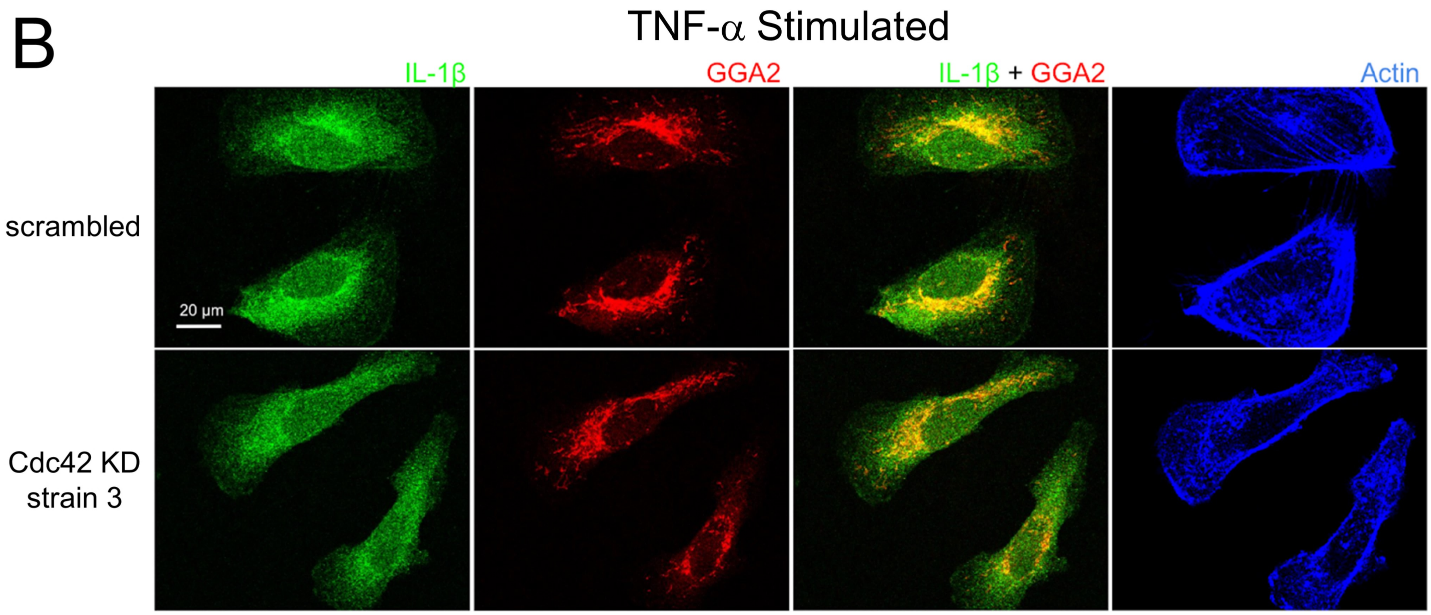

Figure S4 Cdc42-depletion does not affect IL-1β cytosolic trafficking under resting (A) and TNF-α stimulated (B) conditions. BEAS-2B, treated with scrambled control or Cdc42 KD shRNA, were serum-starved overnight, then left unstimulated (*resting*) or stimulated with 10 ng/ml TNF-α for 4 h. Cells were fixed and labeled with antibodies against IL-1β (*green*) and the *trans*-Golgi marker GGA2 (*red*); F-actin was labeled with phalloidin-iFluor 405 (*blue*).
